# Toll-like Receptors in Inborn Errors of Immunity in Children: Diagnostic Potential and Therapeutic Frontiers—A Review of the Latest Data

**DOI:** 10.3390/cells14231902

**Published:** 2025-12-01

**Authors:** Aleksandra Jurczuk, Paulina Bałdyga, Adam Płoński, Maria Jurczuk, Marzena Garley

**Affiliations:** 1 Department of Clinical Immunology, Medical University of Bialystok, 15-274 Bialystok, Poland; 2 Department of Oncological Gynecology with Chemotherapy, Medical University of Bialystok, 15-276 Bialystok, Poland; 3 Department of Vascular Surgery and Transplantation, Medical University of Bialystok, 15-276 Bialystok, Poland; adam.plonski@umb.edu.pl; 4 Department of Toxicology, Medical University of Bialystok, 15-222 Bialystok, Poland

**Keywords:** inborn errors of immunity, Toll-like receptors, MYD88, IRAK4, UNC93B1, TRIF/TICAM1, pediatric, functional diagnostics, genetics diagnostics

## Abstract

Inborn errors of immunity (IEIs), formerly referred to as primary immunodeficiencies (PID), represent a heterogeneous group of hereditary disorders that significantly increase patients’ susceptibility to severe and recurrent infections. Toll-like receptors (TLRs) play a pivotal role in host defense as fundamental components of innate immunity, while also linking it to adaptive immune responses. This review summarizes advances in understanding the involvement of TLRs in the pathogenesis of IEIs in children. It highlights genetic defects such as deficiencies in MyD88, IRAK-4, NEMO, and TLR3, which lead to distinct clinical phenotypes, for example, increased susceptibility to bacterial infections or herpes simplex virus type-1 (HSV-1) encephalitis. The review also examines more complex disorders, including chronic granulomatous disease (CGD), common variable immunodeficiency (CVID), and X-linked agammaglobulinemia (XLA), in which TLR signaling may be either impaired or dysregulated. This analysis demonstrates the growing importance of functional assays evaluating TLR activity as a diagnostic tool complementary to genetic testing, as well as their potential to precisely characterize immunological phenotypes. Furthermore, current therapeutic perspectives are discussed, including the use of TLR agonists, which have shown promising results in oncology, the role of gene therapy as a causal treatment option, and a proposed diagnostic algorithm incorporating TLR-based evaluation. Despite significant progress, substantial knowledge gaps remain, particularly regarding the full spectrum of TLR signaling abnormalities across IEI subtypes. The conclusions emphasize the need for large-scale, international studies to achieve a comprehensive understanding of pathogenic mechanisms and to develop more targeted and effective therapeutic interventions for children affected by these rare disorders.

## 1. Introduction

Inborn errors of immunity (IEIs) represent a heterogeneous group of genetically determined disorders characterized by dysfunction of innate and/or adaptive immune responses [1,2]. Although individual disease entities are rare, as a group they constitute a significant cause of morbidity and mortality, especially in children [3,4,5,6,7]. IEIs are a serious challenge in pediatrics. Clinical symptoms often appear in early childhood and have a broad spectrum, which results in patients presenting to various specialists, including rheumatologists, hematologists, allergologists, and neurologists [1,2,8,9,10].

The diagnosis and treatment of IEIs in children remain a major challenge, particularly in resource-limited countries where access to immunological and genetic testing is insufficient [11]. Early and precise diagnosis is crucial, as it enables the implementation of targeted therapy, including immunotherapy or stem cell transplantation [12].

Toll-like receptors (TLRs) play a key role in innate immunity by recognizing pathogen-associated molecular patterns and triggering cascades of immune responses [13,14]. Disturbances in TLR signaling may lead to susceptibility to severe infections or autoimmune diseases, and therefore they have become the subject of intensive research in the context of IEIs [13,15,16].

The purpose of this article is to provide a concise presentation and critical analysis of the latest literature data concerning the role of TLRs in the pathogenesis of IEIs in children. The analysis focuses on three key areas: (1) the characterization of specific defects in TLR signaling and their correlation with clinical phenotypes, (2) the assessment of the potential use of TLRs in functional diagnostics, and (3) the discussion of new therapeutic and immunomodulatory possibilities.

In the available literature from the last decade, there is a lack of a comprehensive review linking TLR pathway defects in pediatric IEIs with practical diagnostic and therapeutic aspects. This paper identifies key knowledge gaps and proposes potential directions for further research in this field.

The literature review included publications available in PubMed/MEDLINE, Web of Science and Scopus. The following keywords were used: inborn errors of immunity, primary immunodeficiency, pediatrics, Toll-like receptors, TLR3, TLR7, TLR8, MYD88, IRAK4, UNC93B1, TRIF/TICAM1, TBK1, IRF3, IRF7, TRAF3, HSV-1, encephalitis, pyogenic infections, functional diagnostics, whole blood stimulation, fibroblasts, whole genome sequencing, rWGS, interferon, immunomodulation, TLR7/8 inhibitors, IRAK4 inhibitors, juvenile SLE, chilblain lupus, antibiotic prophylaxis. Studies including only animal or cell models without translational potential, commentaries without data, duplicates, publications prior to 2015 (except for key works cited in the text), and conference abstracts without full data were excluded unless they concerned the most recent therapies and were clearly marked as preliminary.

## 2. Mechanisms of Pathogen Recognition by the Immune System

The immune system, whose primary task is to protect the body against pathogens and maintain homeostasis by eliminating damaged or altered host cells, consists of two closely cooperating yet distinct components: innate (nonspecific) immunity and adaptive (specific) immunity [17,18,19,20].

Innate immunity, evolutionarily older, constitutes the first line of defense, providing an immediate response to invading pathogens [21]. The main role of nonspecific immunity is the recognition of Pathogen associated molecular patterns (PAMPs) and Damage associated molecular patterns (DAMPs) originating from injured or dying host cells [10,15]. Identification of PAMPs and DAMPs occurs via Pattern recognition receptors (PRRs), which are germline-encoded and present on cells of the innate immune system such as monocytes/macrophages, neutrophils, dendritic cells, and epithelial cells [10,15]. Among PRRs, Toll-like receptors (TLRs) play a key role in regulating innate immunity. They are considered the earliest determinants of immune system activation [12].

In addition to TLRs, PRRs include RIG-I-like receptors (RLRs)—cytoplasmic sensors of viral RNA that initiate antiviral mechanisms through type I interferon production [22]; C-type lectin receptors (CLRs)—recognizing fungal carbohydrates and bacterial components, enhancing phagocytosis and mediating direct antimicrobial actions [23]; NOD-like receptors (NLRs)—which, upon detecting microbial motifs or stress signals, activate inflammasomes promoting cytokine production and inflammatory responses [23]; and the cGAS-STING pathway—an important defense mechanism against cytoplasmic DNA derived from viruses or damaged host cells, leading to strong activation of interferon-mediated responses [24].

**Figure 1:**
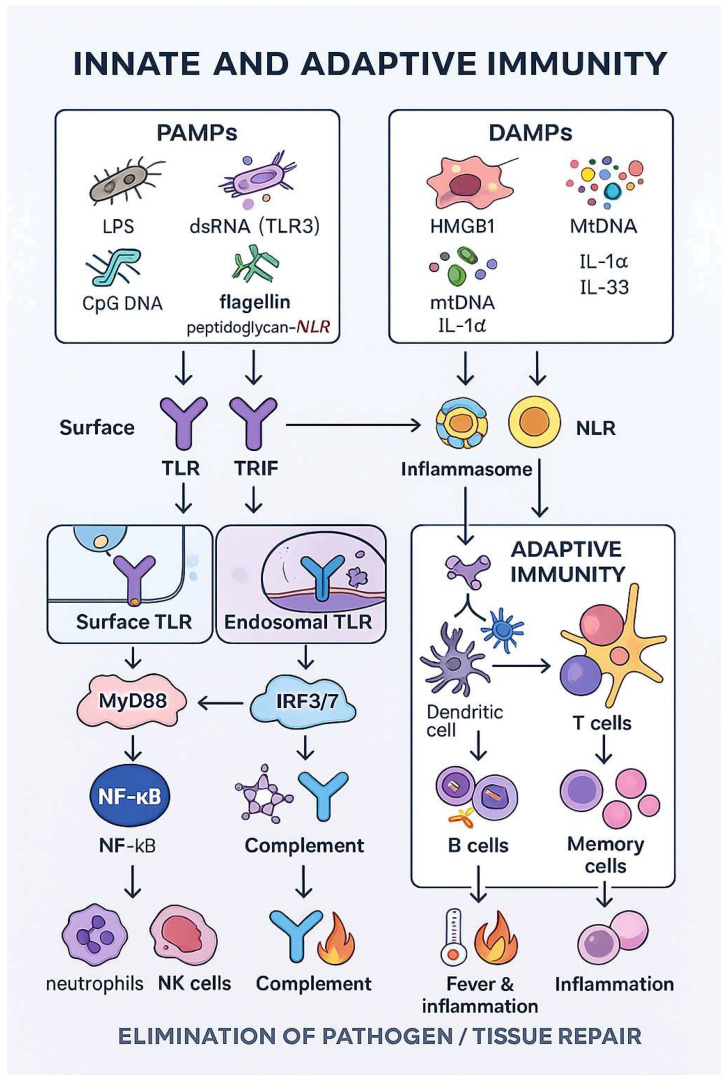
Coordination of innate and adaptive immunity.

Innate immune cells recognize and eliminate pathogenic microorganisms, but they also participate in the activation and expression of the second signal in antigen presentation, which is necessary to initiate adaptive immune mechanisms (Figure 1) [13,14,15,16].

Innate immune responses are initiated when pattern-recognition receptors (PRRs) sense pathogen-associated molecular patterns (PAMPs) and damage-associated molecular patterns (DAMPs). PAMPs include conserved microbial structures such as lipopolysaccharide (LPS), double-stranded RNA, single-stranded viral RNA, flagellin or unmethylated CpG DNA, whereas DAMPs comprise endogenous danger signals released from stressed or dying cells, for example, HMGB1, ATP, urate crystals or mitochondrial DNA. Both TLRs and NLRs can recognize PAMPs as well as selected DAMPs: several TLRs (e.g., TLR2, TLR4, TLR9) respond to endogenous ligands, and some NLRs (e.g., NOD1, NOD2) are activated by bacterial peptidoglycan-derived motifs.

Adaptive immunity requires specialized processes and therefore develops later. The adaptive response involves clonally differentiated T and B lymphocytes, which are characterized by specificity toward particular pathogens and the ability to develop immunological memory [15].

## 3. Toll-like Receptors

**Figure 2:**
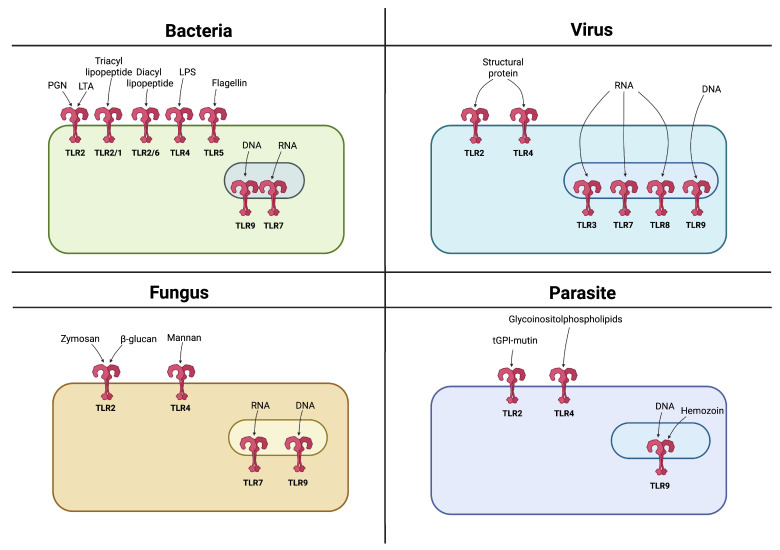
Recognition of PAMPs by TLRs. TLRs are expressed either on the cell surface or within endosomes, with each TLR recognizing specific PAMPs derived from various microbes such as bacteria, viruses, fungi, and parasites. Created in BioRender. Jurczuk, A. (2025) https://BioRender.com/u1bms95 (accessed on 27 November 2025).

TLRs play a particularly important role among PRRs. They recognize PAMPs originating from various microorganisms such as bacteria, viruses, fungi, and parasites, initiating and modulating the course of the immune response (Figure 2) [9,10,11,12].

The name of these receptors derives from the Toll gene, discovered in the 1980s in *Drosophila melanogaster* (fruit fly). Initially, it was believed that this gene regulated the development of the embryonic dorsoventral axis [25]. However, in 1996 it was demonstrated that the Toll gene in the fruit fly plays a fundamental role in antifungal immunity, which laid the foundation for research on analogous proteins in mammals [26].

TLRs are evolutionarily conserved proteins, with the oldest identified in *Caenorhabditis elegans* [27]. Toll-like receptors are characterized by the presence of an extracellular leucine-rich repeat (LRR) domain responsible for ligand recognition. In contrast, intracellularly they possess a domain with high homology to the IL-1R1 receptor (Toll/IL-1 receptor-like, TIR), which enables signal transduction into the cell, activating signaling cascades leading to the production of cytokines, chemokines, type I interferons, and costimulatory molecules [28]. Through this mechanism, TLRs not only initiate an immediate inflammatory response but also serve as a bridge between innate and adaptive immunity by inducing dendritic cell maturation, antigen presentation, and activation of naïve T lymphocytes, which conditions the development of a specific immune response.

### 3.1. Types of Toll-like Receptors

Ten functional TLRs (TLR1–10) have been identified in humans, each specializing in detecting different signals and pathogen-associated molecular patterns [29,30].

**Figure 3:**
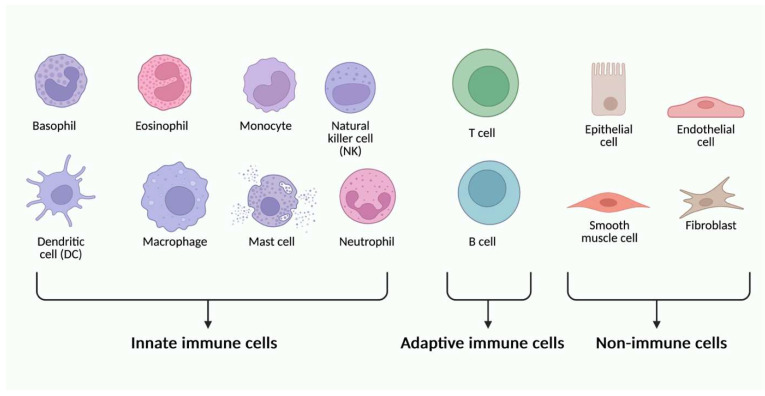
Different types of immune and non-immune cells can express different types of TLR glycoproteins. Created in BioRender. Jurczuk, A. (2025) https://BioRender.com/moegd4t (accessed on 27 November 2025).

TLRs are present mainly on immune system cells, including dendritic cells, macrophages, monocytes, neutrophils, T cells, B cells, mast cells, NK cells, as well as epithelial cells, astrocytes, fibroblasts, keratinocytes, and platelets, and even cancer cells (Figure 3) [30].

The TLR family is functionally diverse—some receptors are located in the cell membrane, while others are found in intracellular compartments such as the endoplasmic reticulum, endosomes, and lysosomes [31].

Surface (membrane) TLRs (TLR1, TLR2, TLR4, TLR5, TLR6, TLR10) recognize PAMPs (e.g., LPS, lipoproteins, flagellin), whereas intracellular endosomal TLRs (TLR3, TLR7, TLR8, TLR9) detect nucleic acids derived from viruses or bacteria [13,26,32,33,34].

Moreover, TLRs are capable of recognizing DAMPs released from damaged or dying cells. DAMPs include extracellular matrix components such as hyaluronan and fibrinogen, plasma membrane elements, nuclear and cytoplasmic proteins such as HMGB1 (*high-mobility group box protein 1*) and heat shock proteins (HSPs), as well as molecules from damaged organelles, e.g., mitochondrial DNA. Upon recognizing DAMPs, Toll-like receptors initiate signaling cascades that activate transcription factors and induce the production of proinflammatory cytokines, type I interferons, and other immune mediators [31,32]. In this way, the immune system can distinguish “self” from “non-self” molecules, thereby maintaining homeostasis and protecting the organism from pathological states such as sterile inflammation or autoimmunity [25].

Intracellular transport of TLRs requires the presence of chaperone proteins (e.g., Unc93B1, gp96, PRAT4A), and some receptors require proteolytic activation, which represents an additional protective mechanism against autoimmunity [35,36].

In recent years, research on TLRs has also focused on their role in regulating immune mechanisms, innate immune training, and their influence on the microbiome and anticancer responses [37,38].

### 3.2. TLRs Signaling Pathways

**Figure 4:**
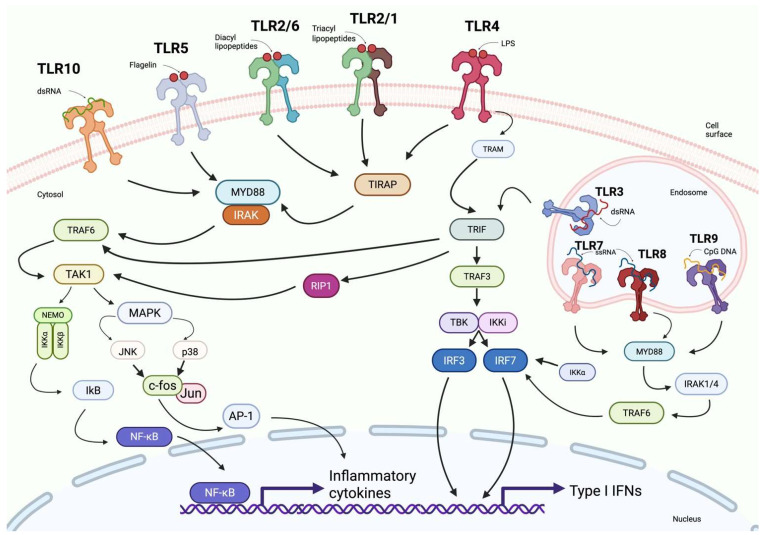
Overview of Toll-like receptor (TLR) signaling pathways. This schematic depicts the cell-surface TLRs (TLR-1, TLR-2, TLR-4, TLR-5, and TLR-6) and their ligands (flagellin, diacyl lipopeptides, triacyl lipopeptides, and LPS). It also shows endosomal TLRs (TLR-3, TLR-7, TLR-8, and TLR-9) recognizing CpG DNA and RNA molecules. The diagram illustrates the subsequent signaling cascades involving adaptor proteins such as MYD88, TRIF, and TRAF6, leading to the activation of NF-κB and IRFs and resulting in the expression of pro-inflammatory cytokines and type I interferons. Created in BioRender. Jurczuk, A. (2025) https://BioRender.com/fahv025 (accessed on 27 November 2025).

Upon recognizing PAMPs or DAMPs, TLRs activate signaling pathways involving adaptor proteins such as myeloid differentiation primary-response protein 88 (MyD88), TIR domain-containing adaptor protein (TIRAP or MAL), TIR domain-containing adaptor protein inducing IFN-β (TRIF), TRIF-related adaptor molecule (TRAM), and sterile α- and armadillo-motif-containing protein (SARM) (Figure 4) [39].

The MyD88-dependent pathway mediates rapid activation of NF-κB and MAPKs downstream of most TLRs, including TLR2, TLR4, TLR5, TLR7, TLR8 and TLR9 [40]. TIRAP activation is dependent on MyD88 and associated with TLR2 and TLR4 [41].

TLR4 recognizes not only the bacterial endotoxin lipopolysaccharide (LPS), but also several endogenous danger-associated molecular patterns (DAMPs), including HMGB1, heme, saturated fatty acids, oxidized phospholipids and extracellular matrix fragments released during tissue injury [42]. After ligand engagement at the plasma membrane, TLR4 signals through the MyD88-dependent pathway, inducing rapid NF-κB and MAPK activation and pro-inflammatory cytokine production. However, following ligand-induced internalization, TLR4 transitions to endosomal compartments, where it initiates a MyD88-independent pathway mediated by the TRAM–TRIF adaptor complex [43].

TRIF signaling recruits TRAF3 together with the kinases TBK1 and IKKε, leading to phosphorylation and activation of IRF3 and IRF7, and subsequent induction of type I and type III interferons, in addition to a delayed NF-κB response [44]. TRAM functions as a bridging adaptor for TRIF in TLR4 signaling, whereas SARM negatively regulates TRIF and modulates TLR3- and TLR4-mediated responses [45].

The key components of the signaling pathway triggered by Toll-like receptors and interleukin-1 receptors (IL-1R) are interleukin-1 receptor-associated kinases (IRAKs). Several IRAK types are distinguished: IRAK-1, IRAK-2, IRAK-3 (also known as IRAK-M), and IRAK-4, of which IRAK-1 and IRAK-4 possess kinase activity, whereas the others serve regulatory roles [46,47,48].

Upon ligand recognition by a TLR, the adaptor MyD88 sequentially recruits IRAK-4 and IRAK-1, leading to the formation of the so-called myddosome—a protein complex that initiates signaling pathways resulting in activation of the transcription factors NF-κB, MAPK, and IRF and subsequent production of pro-inflammatory cytokines, chemokines, and interferons, crucial for an effective immune response [49,50,51,52,53]. IRAK-4, the first kinase activated in the pathway, phosphorylates IRAK-1, which then undergoes autophosphorylation and, together with the adaptor TRAF6, dissociates from the complex. This signal triggers kinase cascades and expression of pro-inflammatory and antiviral genes [54,55].

IRAK-1 also plays a significant role in negative regulation by phosphorylating the adaptor Mal, leading to its ubiquitination and degradation—a mechanism important for controlling TLR2 and TLR4 signal strength [56,57,58].

IRAK-M (IRAK-3), present mainly in monocytes and macrophages, acts as an inhibitor of TLR signaling by blocking the dissociation of IRAK-1 and TRAF6 from the signaling complex, thereby preventing further signal transmission. Recent studies have shown that IRAK-M stabilizes the myddosome structure and contains a death-domain (DD) with unique interaction surfaces, allowing it to modulate large signaling complexes [52,53,54,59].

Table 1 presents the main locations, ligands, signaling pathways, and functions of individual TLRs, highlighting their importance in immune surveillance and disease pathogenesis.

Balanced functioning of TLRs is essential for proper immune system activity. Dysregulation of their activity, whether excessive or insufficient, is closely associated with the pathogenesis of a wide spectrum of immune disorders [66]. On one hand, excessive or inappropriate activation of TLRs may lead to chronic inflammation and the development of autoimmune diseases such as systemic lupus erythematosus (SLE) [67,68,69,70], rheumatoid arthritis (RA) [71,72] or multiple sclerosis (MS) [73,74,75]. On the other hand, defects in TLR signaling pathways may result in inborn errors of immunity, increasing susceptibility to infections, particularly viral and bacterial [2,76,77,78]. In recent years, a significant increase has been observed in studies focusing on the role of polymorphisms in genes encoding TLRs and altered expression of these receptors in relation to disease susceptibility, clinical course, and treatment response [70,79,80,81,82,83,84].

## 4. Inborn Errors of Immunity in Children

Inborn immune disorders comprise a large group of conditions resulting from genetic defects that impair innate and adaptive immunity, cellular and humoral responses, as well as immune regulation [85,86,87]. They may be inherited in a dominant or recessive manner, autosomally or X-linked, and clinical phenotype penetrance may be complete or incomplete. Patients may present with increased susceptibility to a broad or narrow spectrum of infectious diseases, as well as autoimmune, allergic, and/or neoplastic disorders. Since the introduction of next-generation sequencing, the number of identified disorders has been increasing at an unprecedented rate, encompassing not only rare but also more common genetic defects [88]. Advances in molecular genetics and cellular immunology of IEIs have led to the development of innovative preventive and therapeutic strategies [1,87,89,90,91,92,93,94,95,96].

In 2024, the expert committee of the International Union of Immunological Societies (IUIS) for IEIs added 67 new monogenic defects and 2 phenocopies to the classification [1,2,89]. IEIs are rare and thus classified as rare diseases. Initially, rare diseases were defined as those occurring in approximately 1/10,000 to 1/50,000 births. However, due to continuous research progress, identification of new forms of these disorders, and improved definition of clinical phenotypes [97,98,99], the estimated frequency of IEIs is higher, approximately 1/1000 to 1/5000 births [100,101]. As a group, IEIs are a major cause of morbidity and mortality, particularly in pediatric populations [82,102,103].

Clinical manifestations of IEIs in children vary, ranging from recurrent infections to severe recurrent bacterial, viral, fungal, autoimmune infections, and increased susceptibility to malignancies [100,104]. Early diagnosis of IEIs in children often presents a major diagnostic challenge [105]. Due to the variability of symptoms, diverse age of onset, and nonspecific presentation frequently mistaken for common pediatric conditions [100,106].

Defects may involve T lymphocytes, B lymphocytes, NK cells, phagocytes, complement components, as well as abnormalities in pathogen recognition and intracellular signaling pathways [10,33], e.g., involving Toll-like receptors [31,33,107,108].

### 4.1. Dysfunction of TLR Signaling Pathways in IEIs

Among IEIs, dysfunctions of TLR signaling pathways constitute an important, though relatively rare, category of innate immune defects. Each is characterized by a specific clinical phenotype that may lead to severe viral infections and/or immunoregulatory disorders. Including these defects in classification systems and describing them enables improved diagnostics, personalized treatment, and better care for pediatric patients with IEIs [86,109].

#### 4.1.1. Genetic Defects Directly Affecting TLR Signaling Pathways

Identification of monogenic defects that directly impair TLR signaling pathways has enabled fundamental understanding of their significance in the human immune system [85].

MyD88 and IRAK-4 deficiencies

MyD88 and IRAK-4 deficiencies are among the best-characterized defects of innate immunity.

MyD88 deficiency results from autosomal recessive mutations in the *MYD88* gene, which affect the death domain or TIR domain of the MyD88 protein. This disrupts its ability to form a signaling complex with TLRs and to recruit IRAK-4 and IRAK-1 [110,111].

IRAK-4 deficiency is caused by inherited mutations in the IRAK4 gene. The most commonly identified mutations are loss-of-function variants, such as the nonsense mutation p.Q293X, which leads to premature termination of translation and absence of functional protein [112]. These mutations result in complete blockade of signaling dependent on most TLRs (except for TLR3) and IL-1R, preventing activation of NF-κB transcription factors and the production of pro-inflammatory cytokines and interferons [112,113,114].

Patients with these defects are highly susceptible to recurrent invasive bacterial infections, particularly those caused by encapsulated bacteria such as *Staphylococcus aureus* and *Streptococcus pneumoniae* [113,115]. These infections often present with severe clinical manifestations, including sepsis, meningitis, or osteomyelitis, and mortality during the first decade of life may reach 40% [113,115,116]. Early diagnosis is crucial for the implementation of antibiotic prophylaxis, which significantly reduces mortality in children [117,118].

A highly characteristic and diagnostically important sign in these patients is the absence of fever and other classical inflammatory markers (e.g., elevated CRP or ESR), despite active and severe infection [115]. This phenotype directly reflects impaired signaling critical for production of pro-inflammatory cytokines such as TNF-α, IL-6, and IL-1β [115]. Linking the molecular cause (absence of MyD88/IRAK-4) to the observed lack of fever demonstrates a direct causative relationship between the molecular defect and a key clinical feature, which facilitates diagnosis and guides functional testing.

Although MyD88 and IRAK-4 deficiencies block canonical TLR signaling, several studies have reported paradoxically increased accumulation or phosphorylation of IRAK-1 in patient cells. Importantly, this phenomenon does not represent true pathway hyperactivation but results from disrupted negative feedback regulation, which under physiological conditions requires a functional MyD88–IRAK-4 complex. In healthy cells, IRAK-4 phosphorylates IRAK-1, promoting both its activation and subsequent ubiquitin-dependent degradation, which terminates signaling. When MyD88 or IRAK-4 is absent, IRAK-1 cannot be efficiently processed or degraded, leading to its intracellular accumulation in an inactive state, without downstream NF-κB activation. Thus, the apparent “excess activation” reflects defective turnover rather than genuine signaling enhancement, and is directly linked to the molecular consequences of the underlying genetic defect [110,119,120,121].

TLR3, UNC93B1 deficiencies and other TLR signaling defects in antiviral immunity and neuroinfections

Although Toll-like receptors are traditionally associated with antibacterial responses, some of them—particularly endosomal TLRs (TLR3, TLR7, TLR8, and TLR9)—play a key role in viral detection by recognizing viral nucleotides. Impairments in their function lead to significant weakening of antiviral immunity, especially within the central nervous system [111,122].

The most characteristic clinical manifestation of TLR3 deficiency in children is herpes simplex encephalitis (HSE) caused by herpes simplex virus type 1 (HSV-1) [111,123,124]. The direct cause of this deficiency is loss-of-function mutations in the TLR3 gene located on chromosome 4q35.1, which disrupt viral dsRNA recognition and impair activation of the IRF3 pathway and production of type I (IFN-α/β) and type III (IFN-λ) interferons [124,125,126,127]. These mutations may be autosomal dominant or recessive and result in increased susceptibility to viral neuroinfections, mainly HSE [111,123,126]. HSE is associated with high mortality and often leads to severe neurological complications, making early recognition of these deficiencies in children critically important.

A similar phenotype can be observed in patients with mutations in the UNC93B1 gene located on chromosome 11q13.1. This gene encodes a protein responsible for trafficking endosomal TLRs from the endoplasmic reticulum to the endosome, where they become activated by their respective ligands. Mutations in UNC93B1 lead to retention of TLRs in the endoplasmic reticulum and loss of signaling, resulting in defective interferon responses [111,125,128].

TLR5 deficiencies

The TLR5 receptor specifically recognizes flagellin, a protein that forms the flagella of bacteria such as *Salmonella* spp., *Escherichia coli*, *Pseudomonas aeruginosa*, and *Legionella pneumophila* [124,129]. Upon ligand binding, TLR5 activates the MyD88-dependent pathway, leading to NF-κB activation and synthesis of pro-inflammatory cytokines. It is particularly important in the mucosal surfaces of the respiratory and gastrointestinal tracts, which is crucial for natural immunity against flagellated bacteria [130,131].

The direct cause of TLR5 deficiency is the TLR5^392STOP^ mutation. It leads to premature termination of translation, resulting in a nonfunctional protein variant. This defective protein cannot transmit the signal required for flagellin recognition [131]. Individuals carrying the *TLR5*^392STOP^ variant are more susceptible to infections caused by *Legionella pneumophila* [132].

In addition to increased susceptibility to bacterial infections, TLR5 deficiency has been linked to disturbances of mucosal homeostasis and alterations of gut microbiota composition. Experimental models demonstrate that loss of TLR5 results in dysbiosis, impaired epithelial sensing of flagellin, and low-grade intestinal inflammation [132]. Mice lacking TLR5 develop features resembling inflammatory bowel disease (IBD), including enhanced susceptibility to colitis and chronic mucosal immune activation driven by overgrowth of flagellated pathobionts [133]. Moreover, these models reveal that TLR5 deficiency affects systemic metabolic pathways, predisposing to obesity, insulin resistance, and metabolic syndrome through microbiota-dependent mechanisms [133].

Altogether, these findings suggest that TLR5 plays a dual role: first, in direct antibacterial defense via recognition of flagellin, and second, in maintaining gut microbial balance and preventing chronic inflammation. Although human monogenic TLR5 deficiency is rare and not yet well characterized clinically, the strong mechanistic evidence from animal and translational studies justifies its inclusion among innate immune defects with potential relevance to IBD-like and metabolic phenotypes [130].

TLR7 and TLR8 deficiencies

TLR7 and TLR8 are endosomal receptors that recognize single-stranded RNA (ssRNA) from viruses such as SARS-CoV-2, influenza virus (IAV), Zika virus, and Dengue virus [134,135]. Both receptors belong to the same subfamily and are encoded on the X chromosome (Xp22.2 and Xq21.3, respectively). Their activation leads to induction of type I interferons and pro-inflammatory cytokines. These receptors are particularly active in innate immune cells—TLR7 in plasmacytoid dendritic cells (pDCs) and TLR8 mainly in monocytes and macrophages [136,137,138].

TLR7 deficiency results from mutations in the X-linked TLR7 gene. This means the disorder occurs primarily in males, who have only one copy of the gene. This defect was first described in young men with severe COVID-19 who had no prior significant comorbidities. In these patients, germline mutations (e.g., p.Gln710Argfs18* or p. Val795Phe) were identified, leading to impaired TLR7 signaling and loss of type I and III interferon production after stimulation with viral ssRNA. As a consequence, uncontrolled SARS-CoV-2 replication and rapid disease progression occurred [139,140].

TLR8-related disease does not result from loss-of-function but from gain-of-function (GOF) variants. Both germline and somatic mosaic mutations have recently been described—germline mutations are present in all cells of the body, while mosaic mutations arise post-zygotically and are present only in a subset of cells, and the degree of mosaicism correlates with clinical severity, which explains why both males and females may manifest disease. GOF variants cause hyper-responsiveness of immune cells to TLR8 agonists, leading to excessive NF-κB–mediated inflammation. The clinical phenotype includes autoinflammatory symptoms, lymphoproliferation, cytopenias, bone marrow failure, and abnormalities in B- and T-cell development [141,142]. Increased TLR8 signaling also impairs erythropoiesis by inducing inflammatory inhibition of EPO responsiveness, contributing to anemia in many patients [143].

TIRAP deficiency

TIRAP, also known as MAL, is a key adaptor protein involved in Toll-like receptor signaling, mainly TLR2 and TLR4. TIRAP mediates recruitment of MyD88 to the receptor complex by binding its TIR domain, which enables downstream signal transduction leading to NF-κB activation and expression of pro-inflammatory genes [144,145].

The direct cause of this defect is loss-of-function mutations in the *TIRAP* gene that impair protein function. One of the best-described human cases identified an R121W mutation in the TIR domain, which prevents binding to MyD88 and completely blocks TLR2/TLR4-dependent signaling. Functional studies confirmed the defect by demonstrating absent cellular responses to ligands of these receptors [146].

Another variant, D96N, has been identified as a rare polymorphism. It does not disrupt binding to TLR2/TLR4 but prevents interaction with MyD88. These findings show that the D96 residue in the TIR domain is essential for TIRAP–MyD88 binding and proper signal transmission [147,148].

Children with this deficiency present with severe, recurrent bacterial infections—most commonly *Staphylococcus aureus*, *Streptococcus pneumoniae*, and *Haemophilus influenzae*. Symptoms may resemble other defects of the TLR-MyD88-IRAK4 pathway. In contrast to MyD88 or IRAK-4 deficiencies, which cause severe, multi-organ disease, TIRAP deficiency may present with a milder phenotype and partially preserved immune responses, especially against viral infections. Functional studies show that TIRAP-deficient cells have markedly reduced production of pro-inflammatory cytokines such as TNF-α and IL-6 in response to TLR2 and TLR4 stimulation, whereas responses via other TLRs (e.g., TLR3, TLR7, TLR9) may remain relatively intact [78,144].

NEMO syndrome

NEMO syndrome is a rare X-linked recessive immunodeficiency caused by mutations in the *IKBKG* gene, which encodes a key modulator of the NF-κB pathway. NEMO is required to activate NF-κB signaling, which plays an essential role in immunity and development. If NEMO is non-functional, NF-κB cannot enter the nucleus and activate genes necessary for immune responses and cell growth. These mutations often result in ectodermal dysplasia with immunodeficiency (EDA-ID). The EDA-ID phenotype refers to a characteristic combination of developmental abnormalities and impaired NF-κB–dependent immune signaling. Clinically, patients present with features of ectodermal dysplasia such as hypohidrosis or anhidrosis, sparse or absent hair, abnormal or conical teeth, and characteristic skin changes, together with recurrent, severe bacterial, viral, and opportunistic infections resulting from defective activation of NF-κB–mediated inflammatory pathways. This phenotype is most commonly associated with hypomorphic mutations in IKBKG (NEMO), which allow partial NF-κB activity sufficient for survival but inadequate for normal immune, ectodermal, and inflammatory response [115,149].

The disease presents in early childhood. Children with NEMO syndrome suffer from recurrent, severe bacterial infections (e.g., *Streptococcus pneumoniae*, *Staphylococcus aureus*), as well as viral, fungal, and atypical infections—including disseminated mycobacterial disease (e.g., after BCG vaccination) [150,151].

Most patients have the classical EDA-ID phenotype. Others may develop more severe, extended syndromes such as OL-EDA-ID, characterized by osteopetrosis and lymphedema [152].

Cases have also been identified in which heterozygous NEMO mutations cause incontinentia pigmenti (IP) in females, while hemizygous hypomorphic mutations cause EDA-ID in males. Notably, patients with IP generally do not show immunodeficiency [150].

Mechanistically, the difference between IP and EDA-ID arises from the type of NEMO mutation and the pattern of X-chromosome inactivation. Severe loss-of-function mutations in IKBKG are embryonically lethal in males, but heterozygous females survive due to X-inactivation, which selectively removes cells carrying the mutant allele and leaves a population of cells expressing functional NEMO. This skewed X-inactivation protects immune function but results in the characteristic dermatologic manifestations of IP. In contrast, hypomorphic NEMO mutations in males allow survival because some NF-κB activity is preserved, but this residual activity is insufficient for normal immune responses, leading to the combined ectodermal and immunologic defects typical of EDA-ID [152,153].

Diagnosis is based on clinical presentation, immunologic evaluation (low immunoglobulins, elevated IgM, poor vaccine responses), and genetic confirmation of IKBKG mutations [154]. Standard treatment includes immunoglobulin replacement and antibiotic prophylaxis. Hematopoietic stem cell transplantation (HSCT) may correct immune dysfunction but does not resolve ectodermal abnormalities [155].

#### 4.1.2. IEIs in Which TLRs Act as Modulators or Are Indirectly Involved

Common variable immunodeficiency (CVID)

CVID is characterized by the greatest variability of symptoms among these disorders. It is marked by hypogammaglobulinemia and recurrent infections of the upper and lower respiratory tract [156]. Patients are more prone to infections, immune thrombocytopenia, interstitial lung disease, and enteropathy [157,158,159,160]. There are several diagnostic criteria for CVID, but all include low levels of IgG, IgA, or IgM and a poor response to vaccinations [156,161].

TLRs, which are an important group of pattern-recognition receptors, are also involved in the pathogenesis of CVID [162,163]. In patients with CVID, a significantly reduced response of plasmacytoid dendritic cells to TLR7 and TLR9 ligands has been observed. This leads to decreased IFN-α production, which in turn negatively affects B-cell activation and differentiation [164,165,166,167]. Under physiological conditions, activation of these receptors induces B-cell maturation, proliferation, class-switch recombination, and memory-cell formation. In CVID, these processes are impaired, contributing to the characteristic hypogammaglobulinemia and susceptibility to infections [85].

CVID is the most common IEI in adults, but it can also occur in children. Diagnosis in pediatric patients may be difficult due to immaturity of the immune system [168].

Chronic Granulomatous Disease (CGD)

Chronic Granulomatous Disease (CGD) is an inborn error of immunity caused by mutations in the NADPH oxidase complex, leading to impaired generation of reactive oxygen species (ROS) and defective intracellular killing of pathogens. Although TLRs are not directly mutated in CGD, increasing evidence shows that defective ROS production profoundly alters TLR-mediated responses. Neutrophils and monocytes from CGD patients exhibit abnormal expression and function of multiple TLRs, including TLR2, TLR4, and TLR5, resulting in exaggerated or dysregulated cytokine production after stimulation with TLR ligands [169,170,171]. Moreover, ROS act as secondary messengers in TLR signaling pathways, and their absence disrupts normal NF-κB and MAPK activation patterns, contributing to hyperinflammation and granuloma formation [85]. Children with CGD experience recurrent severe bacterial and fungal infections with granuloma formation in organs such as the lungs, liver, and gastrointestinal tract [171].

These findings demonstrate that CGD, although not a classical TLR pathway defect, exhibits significant TLR-related dysregulation, which justifies its inclusion among conditions with impaired or aberrant innate immune signaling.

### 4.2. Epidemiology of TLR-Related Inborn Errors of Immunity

Although TLR-pathway defects constitute a rare subset of inborn errors of immunity, several epidemiological data points have been reported. MyD88 and IRAK-4 deficiencies together represent approximately 1–2% of genetically diagnosed IEIs. Fewer than 80 patients with MyD88 deficiency and fewer than 60 with IRAK-4 deficiency have been described worldwide. These disorders typically manifest in early childhood, and before the introduction of routine antimicrobial prophylaxis, mortality reached 30–40% in early childhood due to invasive pyogenic infections, predominantly caused by Streptococcus pneumoniae and Staphylococcus aureus. Recent data from the COVID-19 pandemic confirmed high vulnerability of these patients to severe viral pneumonia and highlighted the need for aggressive supportive care and prophylaxis [78,172].

TLR3 deficiency is extremely rare, with an estimated prevalence <1 per 1,000,000 individuals. Approximately 40–50 cases have been reported globally. Around half develop herpes simplex encephalitis (HSE), most often during infancy or early childhood. Mortality varies across cohorts, but neurological sequelae are common despite early antiviral treatment [64].

TLR8 gain-of-function (GOF) disease has only recently been recognized. Fewer than 20 cases have been described, including both somatic mosaic and germline variants. The disorder presents in infancy with lymphoproliferation, neutropenia, recurrent infections, B-cell and T-cell dysregulation, and in severe cases, progressive bone-marrow failure. Mortality data remain limited, but severe cytopenias appear associated with poorer outcomes [172,173,174].

NEMO/IKBKG-related ectodermal dysplasia with immunodeficiency (EDA-ID) remains exceptionally rare, with several dozen cases reported. Patients show susceptibility to pyogenic bacteria, mycobacteria and opportunistic infections. Childhood mortality may reach 20–30% without immunoglobulin replacement and prophylaxis [152,174].

## 5. The Role of TLRs in the Diagnosis of IEIs

### 5.1. Functional Screening Tests for TLRs

The diagnosis of IEIs traditionally relies on a comprehensive clinical, immunophenotypic, and genetic evaluation of the patient, allowing for identification of defects in individual components of the immune system [175]. Key diagnostic features of TLR-related inborn errors of immunity summarized in Table 2 provide an overview of the characteristic clinical, immunological, and molecular hallmarks of these disorders, serving as a practical reference for clinicians.

In recent years, functional tests have gained increasing importance in IEI diagnostics, as they allow for direct in vitro assessment of immune cell activity and reactivity to specific immunological stimuli [85]. This approach is particularly valuable when the molecular defect affects signaling pathways activated by PRRs, such as TLRs.

Functional TLR tests have become a key element in the evaluation of innate immune deficiencies, especially in patients with suspected defects in the MyD88 or IRAK-4 signaling pathway, where a lack of or significant reduction in cytokine response after stimulation with TLR agonists is typically observed [113].

Clinical studies have demonstrated that assessing TLR activity enables early detection of subtle innate immune defects, even before they are confirmed by genetic testing [176].

The methodology of TLR testing is based on stimulating peripheral blood mononuclear cells (PBMCs) isolated from the patient with specific ligands for individual receptors. Examples include Pam3CSK4 as a ligand for the TLR1/2 heterodimer, LPS for TLR4, synthetic nucleic acid analogs for TLR3, imiquimod or R848 for TLR7/8, and CpG oligonucleotides for TLR9, used to assess responses to viral or bacterial stimuli [177,178].

After stimulation for a defined period (typically 6–24 h), levels of pro-inflammatory cytokines such as TNF-α, IL-6, IL-1β, and IL-12p70 are measured in the supernatants as markers of TLR/NF-κB pathway activation. Measurements are most commonly performed using ELISA immunoassays, less frequently by flow cytometry or Luminex technology [177,179]. In the case of signaling pathway defects such as IRAK4 or MYD88 mutations, a complete absence of these cytokines is observed, which constitutes a characteristic diagnostic finding [180].

To increase diagnostic accuracy, additional functional readouts are increasingly used. These include assessment of IRAK-1 phosphorylation kinetics, degradation of IκBα as a surrogate of NF-κB activation, CD62L (L-selectin) shedding after TLR4 or IL-1β stimulation, and intracellular cytokine staining in monocytes or dendritic cells. These parameters help differentiate between defects affecting adaptor proteins (e.g., MyD88, TIRAP), kinases (IRAK-4, IRAK-1), or downstream regulators (NEMO) [91].

Several of these assays—especially cytokine-production assays for TLR1/2, TLR4, TLR7/8 and TLR9—are already implemented in specialized clinical immunology laboratories and are routinely performed in expert centers in Europe and the United States as part of the diagnostic workup for suspected TLR-pathway IEIs. Their clinical utility has been validated in cohorts of patients with MyD88, IRAK-4, TLR3, and UNC93B1 deficiencies, where functional defects consistently correlate with molecular diagnosis and clinical phenotype [103,115].

Altogether, functional TLR assays provide a powerful and increasingly standardized tool that complements genetic testing, shortens diagnostic delay, and enables detection of subtle signaling abnormalities that might otherwise remain undetected in routine immunophenotyping.

### 5.2. Proposed Diagnostic Algorithm

To standardize and validate functional TLR assays for clinical use, it is essential to follow a unified methodology. Optimization should include selection of the appropriate material (PBMCs instead of whole blood to eliminate the influence of plasma factors), incubation time, and appropriate ligand concentrations.

The diagnostic algorithm proposed here, based on scientific literature [10,74,85,103,114,173,178,181,182,183,184,185,186], aims to streamline the diagnostic process for this group of defects and shorten the time from clinical suspicion to molecular confirmation. The algorithm combines clinical evaluation, basic immunologic testing, functional innate immunity assays, and genetic diagnostics. A key component is the functional assessment of the TLR/IL-1R pathway, using cytokine assays (IL-6, TNF-α after stimulation with TLR agonists), analysis of IκB-α degradation or IRAK1 phosphorylation, and the L-selectin (CD62L) shedding test.

Combining these tests allows differentiation between specific defects (e.g., MYD88/IRAK4 vs. TLR4 vs. IKBKG) before whole-exome sequencing (WES) is performed.

**Figure 5:**
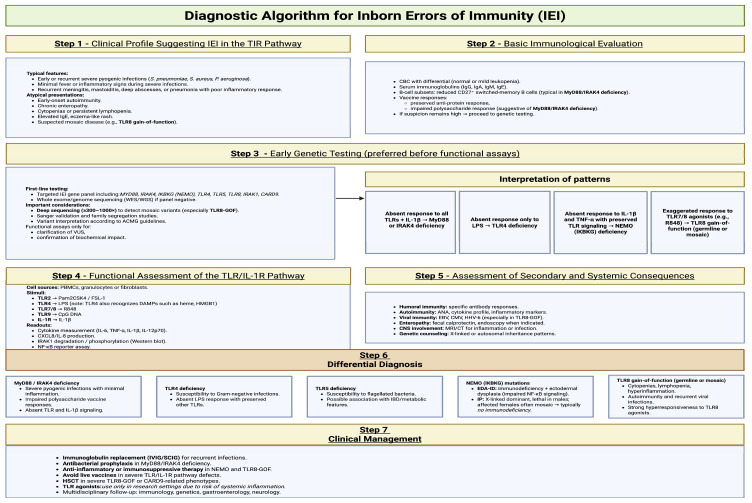
Diagnostic algorithm for IEIs. Created in BioRender. Jurczuk, A. (2025) https://BioRender.com/13onpyv (accessed on 27 November 2025).

Compared with current clinical practice, the proposed diagnostic workflow introduces several key differences and refinements. In most centers, the evaluation of suspected inborn errors of immunity still begins with broad immunological screening followed immediately by genetic testing, while functional innate-immune assays-particularly TLR-specific assays—are rarely performed, are not standardized, and are often used only after genetic findings raise suspicion of a signaling defect. In contrast, the workflow presented in Figure 5 places much greater emphasis on early phenotypic pattern recognition and on the strategic integration of functional assays into the diagnostic pathway. It highlights that specific cytokine-response signatures, IκBα degradation, IRAK1 phosphorylation, or CD62L shedding can rapidly differentiate defects in MyD88/IRAK4, TLR3/UNC93B1, TLR4, or NEMO before sequencing results are available. This contrasts with current practice, where such assays are typically performed only in specialized reference laboratories and are not routinely incorporated into first-line evaluation. The algorithm also departs from standard workflows by recommending early use of deep sequencing (≥300–1000×) when TLR8 gain-of-function mosaicism is suspected, as mosaic variants are frequently missed by conventional whole-exome sequencing. Furthermore, whereas current practice often applies genetic testing without functional contextualization, the proposed approach explicitly integrates clinical phenotype, immunophenotype, functional readouts, and genetic data into a unified interpretation framework, allowing earlier stratification of patients, improved diagnostic precision, and avoidance of diagnostic delay in subtle or atypical IEIs affecting TLR pathways [175,187,188,189,190].

**Table 2:** Key diagnostic aspects of inborn errors of immunity associated with Toll-like receptor pathway dysfunction.

Immunodeficiency/Syndrome	Associated Genetic Defects	Typical Age of Onset	Pathogen Susceptibility/Main Clinical Features	Inheritance	TLR Functional Response	Diagnostic Tests (Functional and Genetic)
IRAK-4/MyD88 deficiency	Mutations in IRAK4 or MYD88	Early childhood (<2 years)	Recurrent invasive bacterial infections (*S. pneumoniae*, *S. aureus*), absence of fever despite severe infection [15].	Autosomal recessive	Markedly reduced TNF-α and IL-6 production after stimulation with TLR ligands (2/1, 2/6, 4, 5, 7, 8, 9).	Functional assays: IL-6, IL-1β, TNF-α production after TLR and IL-1β stimulation. Genetic testing: sequencing of IRAK4 and MYD88 [15].
TLR3 deficiency	Mutations in TLR3, UNC93B1, TRIF	Childhood or adulthood	Recurrent viral encephalitis (HSV-1, VZV, EV-A71) [12].	Autosomal dominant or recessive	Reduced type I IFN (IFN-α, IFN-β) production in response to poly(I:C).	Functional assays: type I IFN induction after poly(I:C) stimulation. Genetic testing: sequencing of TLR3, UNC93B1 [16].
UNC93B1 deficiency	Mutations in UNC93B1	Childhood/adulthood	Severe HSV-1 infection (HSE), susceptibility to VZV and other viruses.	Autosomal recessive	Absent type I IFN response after stimulation via TLR3, 7, 8, 9.	Functional assays: IFN-α/β after stimulation via endosomal TLRs (3, 7, 8, 9). Genetic testing: sequencing of UNC93B1 [191].
TLR5 deficiency	Mutations in TLR5	From childhood to adulthood	Increased susceptibility to *Salmonella*, *P. aeruginosa*; recurrent pneumonia; possible metabolic and autoimmune comorbidities.	Autosomal recessive	Decreased response to flagellin (TLR5 agonist).	Functional assays: response to flagellin. Genetic testing: sequencing of TLR5 [133].
TLR7/TLR8 deficiency	Mutations in TLR7 or TLR8	Early childhood	Severe viral infections (RNA viruses, SARS-tCoV-2) or autoimmune diseases (SLE, autoimmune cytopenias).	X-linked	Diminished or excessive type I IFN production upon TLR7/8 stimulation.	Functional assays: IFN-α/β after R848 stimulation. Genetic testing: sequencing of TLR7, TLR8 [192].
X-linked agammaglobulinemia (XLA)	Mutations in BTK	Infancy	Severe bacterial, viral, and fungal infections [12].	X-linked	Impaired TLR2, TLR4 signaling; in some studies, enhanced responses via TLR4, 7/8, 9.	Functional assays: stimulation with TLR ligands. Genetic testing: sequencing of BTK [193].
Common variable immunodeficiency (CVID)	Often unknown mutations	Adolescence/adulthood	Recurrent bacterial infections of the upper respiratory tract.	Various (often sporadic)	Impaired B-cell and pDC responses to TLR7/9 stimulation; reduced IFN-α secretion.	Functional assays: IL-6 and IFN-α production after TLR7/9 stimulation. Genetic panel sequencing [194].
Chronic granulomatous disease (CGD)	Mutations in NADPH oxidase complex genes [12].	Childhood	Bacterial and fungal infections (especially *Aspergillus*); granuloma formation.	Autosomal recessive or X-linked	Reduced responses via TLR2, TLR4; enhanced responses via TLR5, TLR9.	Functional assays: cytokine production after TLR stimulation; ROS measurement. Genetic testing: sequencing of NADPH oxidase genes [195].
NEMO syndrome (IKBKG)	Mutations in IKBKG	Infancy/early childhood	Severe bacterial, viral, and fungal infections; often ectodermal dysplasia with anhidrosis (EDA).	X-linked	Impaired NF-κB activation upon IL-1β and TNF-α stimulation.	Functional assays: IκBα degradation and NF-κB activation. Genetic testing: sequencing of IKBKG [196].
TRIAP1 deficiency	Mutations in TRIAP1	Not specified	Severe neutropenia, myelodysplastic syndrome.	Autosomal recessive	Not determined.	Genetic testing: sequencing of TRIAP1 [197].

## 6. Therapeutic Strategies Based on TLRs

### 6.1. TLR Agonists as Potential Adjuvants and Therapeutics

The ability to modulate immune responses through TLR stimulation holds enormous therapeutic potential [198]. TLR agonists are being extensively investigated as vaccine adjuvants and in cancer immunotherapy. The aim is to stimulate innate and adaptive immunity to combat cancer cells [199]. An example is the use of TLR7 agonists (e.g., imiquimod, already approved for skin cancer) and clinical trials of TLR9 agonists (CpG oligodeoxynucleotides) in various tumor types [200].

Current treatment strategies for TLR-related inborn errors of immunity (IEIs) depend on the specific genetic defect and the associated pattern of susceptibility. For defects in proximal TLR signaling such as MyD88 and IRAK-4 deficiency, management relies on rapid initiation of broad-spectrum antibiotics, strict clinical monitoring and, in many patients, long-term antimicrobial prophylaxis and immunoglobulin replacement. Recent data from patients with inherited MyD88 and IRAK-4 deficiencies during the COVID-19 pandemic highlight their vulnerability to severe hypoxemic viral pneumonia and emphasize the need for aggressive supportive care and infection prophylaxis [78]. More general treatment overviews for primary immunodeficiencies also stress the central role of anti-infective prophylaxis, immunoglobulin replacement and, in selected cases, hematopoietic stem cell transplantation (HSCT) for severe IEIs [201,202].

In NEMO (IKBKG)-related ectodermal dysplasia with immunodeficiency, treatment is individualized and usually combines immunoglobulin replacement therapy, antimicrobial prophylaxis and aggressive management of bacterial and mycobacterial infections. In selected severe cases with life-threatening infections and profound immunodeficiency, HSCT has been successfully used to correct the immune defect, as illustrated by a 2020 report of treosulfan-based conditioning and allogeneic HSCT in a child with NEMO deficiency [203].

Management of TLR8 gain-of-function (GOF) disease is particularly challenging. Mosaic and germline TLR8-GOF variants define a childhood-onset IEI characterized by lymphoproliferation, neutropenia, recurrent infections, B- and T-cell abnormalities and, in some patients, bone-marrow failure [141]. A focused review has highlighted TLR8-GOF as an “interface” disorder between bone-marrow failure and IEIs, and summarizes current therapeutic experience including antimicrobial prophylaxis, immunoglobulin replacement, immunosuppressive drugs and HSCT [173]. Additional case reports describe severe autoinflammation, autoimmune cytopenias and dysregulation of TLR8/TLR7 signaling [204], while experimental work demonstrates that overexpression or hyperactivation of human TLR8 can induce inflammatory bone-marrow failure and anemia [205]. More recently, a female patient with TLR8 GOF has been reported, underlining that both males and females can be affected and further supporting the role of somatic mosaicism [185]. Collectively, these data suggest that immunosuppressive agents (e.g., corticosteroids, IL-1 blockade, JAK inhibitors) may provide partial control of hyperinflammation, whereas HSCT remains the only potentially curative option in severe TLR8-GOF.

TLR agonists and antagonists are also being actively explored as immunomodulatory drugs in oncology and infectious diseases. Recent reviews and clinical trial overviews emphasize both their potential and the limitations related to systemic inflammation and toxicity. For example, broad analyses of TLR agonists in cancer highlight significant challenges in translating preclinical efficacy into clinical benefit and caution that systemic administration may lead to considerable adverse effects [206,207,208]. In the context of monogenic TLR-pathway IEIs, these safety concerns are even more pronounced, and TLR-targeted drugs remain purely experimental. At present, evidence-based treatment of TLR-related IEIs therefore relies primarily on optimized anti-infective management, immunoglobulin replacement when indicated, targeted immunomodulation in hyperinflammatory phenotypes (such as TLR8-GOF), and HSCT reserved for selected severe cases.

The mechanism of action of TLR agonists—strengthening a weakened immune response—appears particularly promising in the context of IEIs. Theoretically, TLR agonists could compensate for the absence of key proteins in signaling pathways, such as MyD88 or IRAK-4, restoring a patient’s ability to fight infections [209]. Although most research on TLR agonists concerns oncology, their success in this field provides a solid basis for considering their use in IEIs, especially in defects with impaired signaling. Research in this area remains at an early stage but represents a promising and under-explored therapeutic frontier [15]. More recent data also show that patients with inherited MyD88 or IRAK-4 deficiency are at increased risk of severe hypoxemic COVID-19 pneumonia, underlining the need for close monitoring and aggressive supportive care during viral infections [78]. For defects affecting antiviral TLR pathways, such as TLR3 and UNC93B1 deficiencies, early recognition and treatment of herpes simplex virus (HSV) infections are crucial to prevent HSV encephalitis. Case reports and small series in TLR3-deficient patients consistently emphasize prompt initiation of intravenous acyclovir and prolonged treatment courses to reduce relapse and neurological sequelae.

### 6.2. Gene Therapy and Other Treatment Approaches

Gene therapy is a potential therapeutic method aimed at introducing a correct copy of a defective gene into patient cells ex vivo (typically hematopoietic stem cells, HSCs) or correcting the mutation in situ using CRISPR/Cas9, TALEN, or ZFN, followed by autologous transplantation of the modified cells [209,210,211]. In some cases, it is already available for IEI treatment [212]. Successful examples include gene therapy for X-linked severe combined immunodeficiency (SCID-X1) and X-linked chronic granulomatous disease (X-CGD) [213,214,215]. In the context of TLR defects, gene therapy could restore proper signal transduction by supplying a functional copy of IRAK4 or MYD88, offering a permanent and causal treatment solution. This approach is particularly important given the lack of specific targeted drugs for many TLR-associated IEIs.

### 6.3. Clinical Trials in Children

In pediatrics, a key milestone is a phase I clinical trial of a TLR9 agonist (GNKG168) in children with acute lymphoblastic leukemia (ALL) with minimal residual disease [216]. The study demonstrated that administration of the TLR9 agonist was associated with immunologic changes, including immune cell activation and inhibition of immune checkpoint pathways [216]. Although the trial involved oncologic patients, its results provide direct evidence that TLR agonists can be safely and successfully used in children, inducing desired immunomodulatory effects [216]. This opens the door to further research on TLR agonists as adjuvants or targeted therapies for other pediatric diseases, including IEIs, where enhancing a weakened immune response is required.

## 7. Knowledge Gaps and Future Perspectives

### 7.1. Unmet Research Needs

Despite progress in understanding the role of TLRs in IEIs, many gaps remain. A lack of comprehensive studies examining TLR signaling abnormalities in specific IEI subtypes remains a major challenge [209]. Available data are often fragmented or contradictory, particularly regarding the role of TLR pathways in disorders traditionally not classified as TLR-related. A clear example is X-linked agammaglobulinemia (XLA), where some studies report largely preserved TLR responses in monocytes and dendritic cells, while others describe significant alterations in TLR-induced cytokine production and impaired downstream signaling [85,217]. Another critical issue is global inequality in care for patients with IEIs, strongly correlated with a country’s Human Development Index (HDI) [11]. In many regions, basic immunological and genetic tests are unavailable, and diagnosis relies on simple clinical criteria, preventing the implementation of precise and effective therapies [11]. Additionally, challenges persist in translating promising preclinical and oncology-derived findings into clinical practice for IEIs. This requires costly and highly specific clinical trials, which is particularly difficult for rare diseases.

### 7.2. Development Perspectives

Future research on TLRs in IEIs should focus on several key areas. Large, international multicenter studies are needed to enroll sufficiently large cohorts of patients with rare IEIs. Global collaboration will allow comprehensive phenotypic characterization and evaluation of therapeutic responses, which is impossible in single-center studies [11]. The feasibility of such efforts is supported by existing international networks, including the European Society for Immunodeficiencies (ESID) Registry, the USIDNET registry in North America, and global research consortia such as the International Union of Immunological Societies (IUIS) IEI Committee, which have already enabled cross-country patient aggregation and collaborative studies in rare IEIs [218,219,220].

Another priority is the development of accessible, standardized, and cost-effective functional TLR diagnostic tests that could become part of routine IEI diagnostics. Further efforts should also focus on identifying new TLR agonists and antagonists that could target specific innate immune defects [85]. In deficiencies with impaired TLR responses, agonists may help stimulate cytokine production and restore immune function. Conversely, in disorders with excessive signaling, antagonistic molecules may help restore immune homeostasis.

Finally, the future of IEI treatment lies in the advancement of gene therapy. As gene-editing technologies evolve and improve, precise and safe correction of genetic defects becomes possible, potentially curing a growing number of patients with TLR-related IEIs [221].

## 8. Conclusions

The literature confirms the crucial and multifaceted role of TLRs in the pathogenesis of IEIs in children. From monogenic defects with predictable clinical phenotypes to complex functional abnormalities in more common IEIs, TLR signaling dysfunction underlies many of these disorders. Functional TLR testing is a valuable diagnostic tool that allows assessment of molecular defects and supports accurate diagnosis and effective treatment. The therapeutic potential associated with TLR modulation is substantial, and the success of TLR agonists in oncology provides strong justification for exploring their use in IEIs.

In the future, priorities should include deepening the understanding of signaling mechanisms, standardizing diagnostics, and developing and testing targeted therapies. The ultimate goal is to transition from symptomatic to causal treatment, enabled by precise targeting of molecular defects in innate immunity. Integrating knowledge about TLRs with advancements in genetics and gene therapy opens the path toward more effective and personalized treatment for children with inborn errors of immunity.

## Figures and Tables

**Table 1:** Toll-like receptors—localization, ligands, signaling pathways, and functions.

TLR	Localization	Main Ligand	Signaling Pathway	Functions and Example Applications
TLR1/2	Cell surface	Triacylated lipopeptides (Pam3CSK4)	MyD88-dependent	Activation of immune response; potential applications in cancer and infectious disease therapy [28]
TLR2/6	Cell surface	Lipopeptides	MyD88-dependent	Regulation of inflammatory processes and immune responses; potential therapies for allergic and autoimmune diseases [60]
TLR3	Endosomes	Double-stranded RNA (dsRNA)	TRIF-dependent	Antiviral response; applications as a vaccine adjuvant and in cancer immunotherapy; requires UNC93B1 [61]
TLR4	Cell surface	Lipopolysaccharide (LPS), HMGB1; heme; saturated fatty acids; oxidized phospholipids; ECM fragments	MyD88- and TRIF-dependent	Regulation of inflammatory response; potential applications in treating infections and cancers; requires coactivator MD2 and CD14 [62]
TLR5	Cell surface	Flagellin	MyD88-dependent	Recognizes bacterial flagellin, regulates gut homeostasis and microbiota; promotes mucosal immunity. Flagellin is used as a potent vaccine adjuvant and mucosal immunostimulant. Role in metabolic regulation and aging [63,64]
TLR7	Endosomes	Single-stranded RNA (ssRNA)	MyD88-dependent	Antiviral response; regulation of autoimmune diseases such as lupus (SLE); type I IFN activation [65]
TLR8	Endosomes	Single-stranded RNA (ssRNA)	MyD88-dependent	Antiviral and anticancer responses; therapeutic applications; similar role to TLR7; activates dendritic cells [65]
TLR9	Endosomes	CpG DNA	MyD88-dependent	Antiviral response; applications as a vaccine adjuvant and in treatment of infections and cancers; requires proteolysis [60]
TLR10	Cell surface	Double-stranded RNA (dsRNA)—presumed	MyD88-dependent	Potential role in antiviral response and immune regulation; requires further research [60]

## Data Availability

No new data were created in this study.
